# PAI1 Regulates Cell Morphology and Migration Markers in Trastuzumab-Resistant HER2-Positive Breast Cancer Cells

**DOI:** 10.3390/life14081040

**Published:** 2024-08-20

**Authors:** Asiye Busra Boz Er, Idris Er

**Affiliations:** 1 Department of Medical Biology, Faculty of Medicine, Recep Tayyip Erdogan University, Rize 53200, Turkey; asiyebusra.bozer@erdogan.edu.tr; 2 Department of Medical Biology, Faculty of Medicine, Karadeniz Technical University, Trabzon 61080, Turkey

**Keywords:** breast cancer, HER2, metastasis, morphology, PAI1, TGF-β

## Abstract

HER2-positive breast cancer is a significant cause of mortality. Overcoming trastuzumab resistance requires a deeper understanding of its molecular mechanisms to develop effective therapies. This study investigates the role of plasminogen activator inhibitor-1 (PAI1) in migration and drug resistance in trastuzumab-resistant HER2-positive breast cancer. Trastuzumab resistance poses a significant challenge in clinical management due to its association with aggressive disease behaviour and limited treatment options. This study focuses on PAI1, a key player in the TGF-β signalling pathway, which is implicated in cancer progression and metastasis. Trastuzumab-resistant cell lines (SKBR3 and HCC1954) demonstrated markedly elevated PAI1 expression levels, up to 40-fold compared to parental lines. This elevation was accompanied by increased expression of migration markers such as Col4a1, Fibronectin, ICAM1, Timp2, and Vimentin. Through overexpression and silencing experiments, we observed that modulating PAI1 levels significantly impacts cell morphology, transitioning cells from an epithelial to mesenchymal phenotype. Importantly, combining trastuzumab with aleplasinin, a PAI1 inhibitor, synergistically reduced PAI1 expression in both parental and resistant cell lines. This suggests a potential therapeutic strategy to overcome trastuzumab resistance. These findings emphasise PAI1 as a critical mediator of migration and therapeutic response in HER2-positive breast cancer, offering insights into novel treatment approaches targeting PAI1 to improve clinical outcomes in drug resistance.

## 1. Introduction

Breast cancer is a leading cause of cancer-related deaths among women worldwide, with 15–20% of cases being human epidermal growth factor receptor 2 (HER2)-positive [1]. This subtype is characterised by the amplification of the HER2, which results in aggressive tumour behaviour and poorer prognoses [2]. Trastuzumab, a monoclonal antibody targeting HER2, has significantly improved survival rates and the quality of life for patients [3]. However, the emergence of resistance to trastuzumab poses a major challenge in long-term management [4]. Resistance to trastuzumab can arise through multiple molecular mechanisms, underscoring the complex nature of HER2 signalling and its interplay within cancer cells [5]. Key mechanisms implicated in trastuzumab resistance include alterations in HER2 expression levels, downstream signalling pathway activation (such as PI3K/AKT/mTOR/Hedgehog pathway), and dysregulation of apoptotic pathways (e.g., Bcl-2 family proteins) [5,6]. Additionally, crosstalk with other receptor tyrosine kinases (RTKs) and activation of alternative survival pathways contribute to therapeutic resistance in HER2-positive breast cancer.

Research into new treatments for trastuzumab resistance is driving novel strategies. These include the development of next-generation HER2-targeted therapies (e.g., pertuzumab, T-DM1), combination therapies targeting alternative signalling pathways (e.g., PI3K inhibitors), and immunotherapeutic approaches (e.g., antibody–drug conjugates) [7]. Moreover, strategies focusing on tumour microenvironment modulation, such as inhibition of angiogenesis and immune checkpoint blockade, are being explored to enhance treatment responses in resistant HER2-positive breast cancers.

One of the mechanisms implicated in trastuzumab resistance is the activation of the TGF-β signalling pathway [8]. Transforming growth factor-beta (TGF-β) is a multifunctional cytokine that regulates cell proliferation, differentiation, and migration. TGF-β and HER2 signalling work together to drive breast cancer progression. However, the details of how these two pathways interact at the molecular level are not well understood. This lack of clarity makes it challenging to develop better treatments [9]. Previous studies have identified several TGF-β responsive genes that contribute to cancer progression, including *WWP1*, *CARM1*, *RASGRP1*, *THBS1*, *MMP2*, and *MMP9* [10,11]. In this study, by analysing previous works, we aim to investigate these responsive genes in HER2-positive trastuzumab-resistant cell lines.

Plasminogen activator inhibitor-1 (PAI1) is a key regulator of the TGF-β induced cell response and has been implicated in cancer cell migration and metastasis [12]. PAI1 modulates extracellular matrix (ECM) remodelling and influences cell motility by interacting with matrix metalloproteinases (MMPs) [13]. Elevated PAI1 expression has been associated with poor prognosis in various cancers, including breast cancer. In HER2-positive breast cancer cells, PAI1 expression is prominently elevated, indicating its potential involvement in resistance mechanisms and increased migratory capabilities [14]. However, its precise role in both HER2-positive breast cancer and trastuzumab-resistant HER2-positive breast cancer remains poorly understood.

This study investigates the role of PAI1 in regulating migration and response to therapy in trastuzumab-resistant HER2-positive breast cancer cell lines SKBR3 and HCC1954. The expression of TGF-β responsive genes and migration markers is analysed in parental and resistant cell lines. Additionally, the effects of PAI1 overexpression and silencing on cell morphology and migration are explored. Finally, the impact of combining trastuzumab with a PAI1 inhibitor, aleplasinin, on gene expression is assessed. Insights into the molecular mechanisms underlying trastuzumab resistance are provided, suggesting potential therapeutic strategies to overcome resistance in HER2-positive breast cancer.

## 2. Materials and Methods

### 2.1. Cell Culture, Transfection, Drug Treatment

HCC1954 (ATCC Cat#CRL2338) and SKBR3 (ATCC Cat#HTB30) are HER2-positive breast cancer cell lines purchased from ATCC. These cell lines are known for their capability to develop trastuzumab resistance [15,16]. The cell lines were grown in DMEM media supplemented with 10% FBS, 1% sodium pyruvate, and 2 mM L-glutamine. HCC1954 and SKBR3 trastuzumab-resistant cell lines were developed by treating the cells with increasing doses (initial dose 0.1 and terminated dose 10 μM) of trastuzumab over a period of 3 months. To verify the acquisition of resistance in these newly generated cell lines, MTT viability assays were conducted, and the IC50 values were analysed according to the methods outlined in our previous study [17]. Chronic exposure to trastuzumab led to the development of resistance, resulting in elevated IC50 levels. The IC50 values for trastuzumab were 0.2 μM in SKBR3-P, 2.6 μM in SKBR3-R, 0.3 μM in HCC1954-P, and 2.4 μM in HCC1954-R. Details on IC50 levels for aleplasinin can be found in Table 1. For further experimentation, HCC1954 and SKBR3 cells were seeded into six-well plates at a density of 2.2 × 10^6^ cells per well, with a confluency of 40–60%. After 24 h, the cells were transfected with 2 μg of plasmid DNA per well using lipofectamine 2000 [Waltham, MA, USA], following the manufacturer’s instructions.

### 2.2. Combination Therapy Experiment

SKBR3-P, SKBR3-R, HCC1954-P, and HCC1954-R cells were treated with trastuzumab and aleplasinin in a 1:1 concentration ratio at 0.25-fold, 0.5-fold, 1-fold, 2-fold, and 4-fold of the individual IC50 values (see Table 1). The combination effects were assessed using an MTT assay. The CompuSyn software 1.0 was employed to calculate the combination index (CI), which indicates the interaction between the drugs. A CI > 1.1 indicates antagonism, a CI < 0.9 suggests synergy, and a CI between 0.9 and 1.1 denotes an additive effect [18].

### 2.3. Quantitative Real-Time PCR

RNA isolation was performed using the Qiagen RNAeasy kit following the manufacturer’s guidelines. cDNA synthesis was carried out using Biorad iScript Reverse Transcription Supermix for RT-qPCR. PCR was conducted with the iTaq Universal SYBR Green One-Step Kit, and Ct values were measured with the Applied Biosciences ABI 7500 Real-Time Instrument and 7500 Software v1. The amplification conditions were set at 95 °C for 10 s and 60 °C for 1 min over 40 cycles; the melting curve consisted of denaturation at 95 °C for 15 s, annealing at 60 °C for 1 min, and elongation at 95 °C for 15 s. Real-time PCR primers, designed using the Primer3 program and provided by Macrogen (see Table 2), were used. Each PCR run was performed in triplicate as technical repeats, and the experiments were repeated three times with different samples. RT-qPCR data analysis involved normalising the cDNAs’ threshold cycle (Ct) values to the housekeeping gene GAPDH. Data were calculated using the following formula: average of technical repeats and ΔCt = Ct (average of target gene) − Ct (average of housekeeping gene). Fold changes were determined by calculating 2^−ΔCt^ and 2^−ΔΔCt^:2^−ΔCt^ (sample)/2^−ΔCt^ (control).

### 2.4. Gene Expression Heatmap

Heatmaps were created using the Python programming language, with libraries including NumPy, Matplotlib, and Seaborn. The process was conducted in Google Colab, which provided an interactive environment for executing the Python code. Data consisting of gene names (as row labels), cell line names (as column labels), and corresponding quantitative values were organised into a NumPy array. The Seaborn library’s heatmap function was used to visualise this data matrix, with a diverging colour palette chosen to distinctly represent positive and negative values. Axes were labelled appropriately, and the resulting heatmap was generated and displayed using Matplotlib within the Google Colab environment.

### 2.5. Western Blot

Cells were lysed in TNTE (Tris-NaCl-Triton X-100-EDTA) buffer. For protein separation, 20 µg of samples were loaded onto a 7.5% acrylamide gel with 10% SDS running buffer. Precision Plus Protein™ Dual Color Standards (Bio-Rad [Hercules, CA, USA]) served as molecular weight markers. Proteins were transferred to a PVDF membrane, which had been pre-soaked in methanol, using 1X cold transfer buffer on ice.

The membranes were blocked with 5% skimmed milk in TBST. Primary antibodies—Mouse monoclonal PAI1 (sc-5297, Santa Cruz [Santa Cruz, CA, USA]) and Mouse anti-α-Tubulin (Sigma [Burlington, MA, USA])—were diluted to 1/1000 in 5% skimmed milk in TBST and incubated overnight at 4 °C. The secondary antibody, Goat Anti-Mouse IgG-HRP (Santa Cruz), was applied at a dilution of 1/7500 in 5% skimmed milk in TBST and incubated for one hour. Proteins were detected using Clarity™ Western ECL Substrate (Bio-Rad [Hercules, CA, USA]) and visualised with the Bio-Rad Chemidoc MP. Band intensity was quantified using Image Lab (6.1.0, built 7) (Bio-Rad, Hercules, CA, USA) software.

### 2.6. Immunofluorescence

Sterile glass slides were placed in 6-well plates, and 2.2 × 10^5^ cells were seeded into each well. After 24 h, the cells were transfected with SERPINE1-bio-His (a plasmid for PAI1 overexpression, provided by Gavin Wright, Addgene plasmid #52077) [19] and pcDNA3.1-6XHis (a control plasmid) using Lipofectamine 2000, with a 24 h incubation period. Transfection was carried out using siPAI1 (Horizon Discovery, cat no: L-019376-01-0010) and siCTRL (Horizon Discovery-Dharmacon [Lafayette, CO, USA]) with Lipofectamine™ RNAiMAX Transfection Reagent (Cat no: 13778075). After transfection, cells were fixed in methanol for 10–15 min, permeabilised with 0.2% Triton X-100 in PBS, and blocked for 10 min to 1 h in 10% bovine serum albumin (BSA)–PBS. The cells were then incubated for 2 h with the primary antibody Mouse anti-β-Actin (Sigma) at a 1:500 dilution in 10% BSA–PBS, followed by a 1 h incubation with the secondary antibody Goat Anti-Mouse IgG-Alexa Fluor 488 at a 1:100 dilution. Finally, the cells were mounted with DAPI.

### 2.7. Statistical Analysis

Statistical analysis was performed using a two-tailed Student’s *t*-test, two-way ANOVA, and Tukey’s post hoc test to assess significance, with a *p*-value of * ≤ 0.05 deemed significant. Error bars represent ± SD from three independent experiments, each conducted in triplicate. Details of the analytical methods and *p*-values are indicated in the figure legends.

## 3. Results

### 3.1. TGF-β and Migration Responsive Genes Regulated by Acquired Resistance in HER2-Positive Trastuzumab Resistant Cell Lines

It is known that TGF-β has an increase in HER2-positive trastuzumab-resistant cell lines. WWP1, CARM1, RASGRP1, THBS1, KCTD5, SGCA, EIF3S6, MCAM, FXR2, MTMR3, SOCS3, SLC2A4RG, THBS1, MMP2, MMP9, HSP47, PAI1, TIMP1, TGIF, COL6A1, AAK1, MAN1A1, NT5C2, IRS2, CSEN, C4BPA, PSG1, ALAD, FGD1, TFAP2B, MIDORI, AHCYL1, HSPG2, IL1RAP, and MAN1A1 TGF-β responsive genes have been determined in A549 (lung adenocarcinoma) and HPL1D (lung epithelial cell line) [10], but the same genes have not been analysed in HER2-positive trastuzumab resistant cell lines. In our study, we analysed the genes referenced in their work and other known TGF-β responsive genes, MMP2, MMP9, HSP47, PAI1 [11] and TIMP1, TGIF, COL6A1 [20].

**Figure 1:**
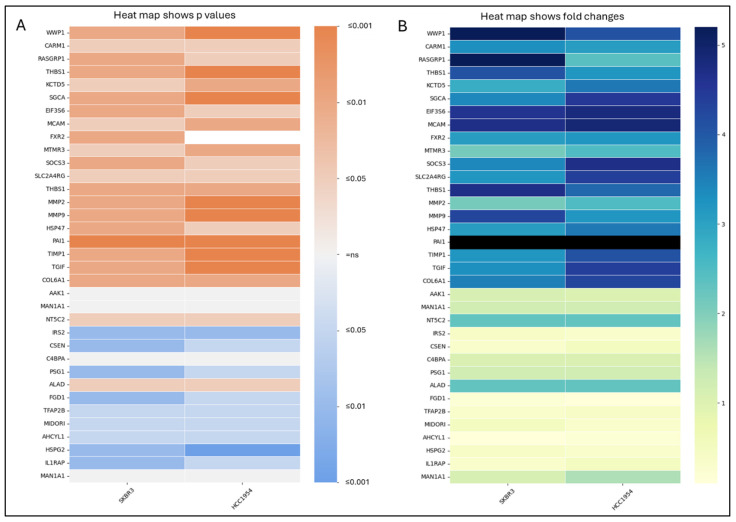
Heat map visualisation of the normalised gene expression levels for TGF-β responsive genes for p-value significance score and fold changes: (**A**) Heatmap shows *p* values of selected gene expressions. Orange shows the *p*-values belonging to increased gene *p* values; blue belongs to decreased gene *p* values; white colour shows ns = non-significant. (**B**) The heatmap shows fold changes in selected gene expressions. Blue shows the increases, and yellow (below 1) shows decreases. Parental cells are used as the experimental control. GAPDH is used as an expression control. A *p*-value is below 0.05, determined by Student’s *t*-test, n = 3.

It was observed that WWP1, CARM1, RASGRP1, THBS1, KCTD5, SGCA, EIF3S6, MCAM, FXR2, MTMR3, SOCS3, SLC2A4RG, THBS1, MMP2, MMP9, HSP47, PAI1, TIMP1, TGIF, COL6A1, NT5C2, and ALAD was significantly increased, and IRS2, CSEN, PSG1, FGD1, TFAP2B, MIDORI, AHCYL1, HSPG2, and IL1RAP was significantly decreased in SKBR3 resistant cells; WWP1, CARM1, RASGRP1, THBS1, KCTD5, SGCA, EIF3S6, MCAM, MTMR3, SOCS3, SLC2A4RG, THBS1, MMP2, MMP9, HSP47, PAI1, TIMP1, TGIF, COL6A1, NT5C2, and ALAD was significantly increased, and IRS2, CSEN, PSG1, FGD1, TFAP2B, MIDORI, AHCYL1, HSPG2, and IL1RAP was significantly decreased in HCC1954 resistant cell lines (Figure 1A).

WWP1, CARM1, RASGRP1, THBS1, KCTD5, SGCA, EIF3S6, MCAM, FXR2, MTMR3, SOCS3, SLC2A4RG, THBS1, MMP2, MMP9, HSP47, PAI1, TIMP1, TGIF, COL6A1, NT5C2, and ALAD has fold changes belonging to increased gene expressions, and IRS2, CSEN, PSG1, FGD1, TFAP2B, MIDORI, AHCYL1, HSPG2, IL1RAP has fold changes belonging to decreased gene expressions in SKBR3 resistant cells; WWP1, CARM1, RASGRP1, THBS1, KCTD5, SGCA, EIF3S6, MCAM, MTMR3, SOCS3, SLC2A4RG, THBS1, MMP2, MMP9, HSP47, PAI1, TIMP1, TGIF, COL6A1, NT5C2, and ALAD has fold changes belonging to increased gene expressions and IRS2, CSEN, PSG1, FGD1, TFAP2B, MIDORI, AHCYL1, HSPG2, IL1RAP has fold changes belonging to decreased gene expressions in HCC1954 resistant cell lines (Figure 1B).

**Figure 2:**
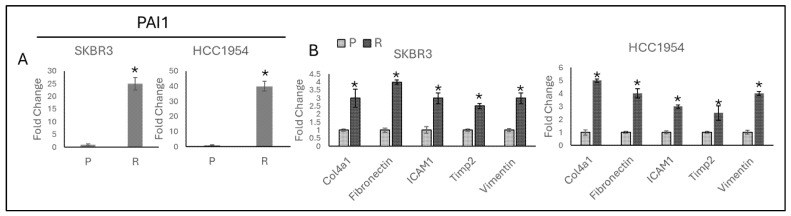
Acquired trastuzumab resistance increases *PAI1* gene expression and migration markers in parental and resistant SKBR3 and HCC1954 cell lines. (**A**) Approximately a 25-fold increase was observed in HCC1954, and a 40-fold increase was observed in SKBR3-resistant cell lines. (**B**) *Col4a1*, *Fibronectin*, *ICAM1*, *Timp2*, *Vimentin* gene expressions were significantly increased in SKBR3 and HCC1954 cell lines resistant cell lines. P: parental, R: resistant. A two-tailed Student’s *t*-test was used. * *p* ≤ 0.05, n = 3 ± SD.

Interestingly, the high fold change was observed in *PAI1* levels in both resistant cell lines. PAI1 increased 25-fold in SKBR3 and 40-fold in HCC1954-resistant cells (Figure 2A). Beyond the role of *PAI1* expression as a TGF-β responsive gene, it was also known that *PAI1* modulates matrix remodelling and migration in HACAT cells [21], but its role in HER2-positive cells was unknown.

*Col4a1*, *Fibronectin*, *ICAM1*, *Tip2*, and *Vimentin* are migration-responsive genes [22], and their expression is analysed in resistant cells. It was observed that *Col4a1*, *Fibronectin*, *ICAM1*, *Tip2*, and *Vimentin* migration marker genes were significantly increased in both SKBR3- and HCC1954-resistant cell lines (Figure 2B).

### 3.2. PAI1 Regulates Migration Responsive Gene Expressions in Parental and Resistant HER2-Positive Cell Lines

**Figure 3:**
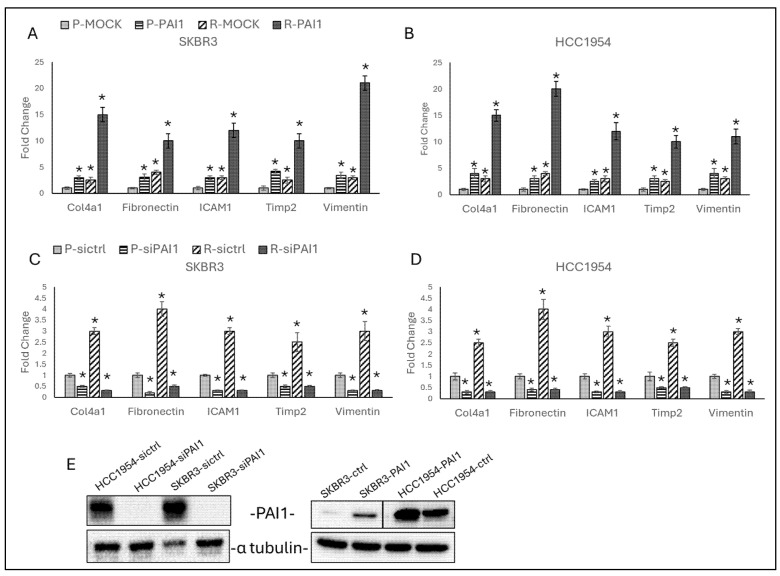
PAI1 regulates migration markers in HCC1954 and SKBR3 parental and resistant cells: (**A**) Overexpression of PAI1 in SKBR3 and (**B**) HCC1954 parental and resistant cells increased migration marker gene expressions. (**C**) Silence of PAI1 in SKBR3 and (**D**) HCC1954 parental and resistant cells decreased migration marker gene expressions. (**E**) Silence and overexpression of PAI1 in HCC1954 and SKBR3 cells. P: parental, R: resistant. Two-way ANOVA variation test and Tukey post hoc test were used. * *p* ≤ 0.05, n = 3 ± SD.

To observe the effect of PAI1 expression on migration in trastuzumab-resistant cells, PAI1 was overexpressed and silenced in SKBR3 and HCC1954 parental and resistant cell lines, and protein levels were analysed by Western blot (Figure 3E).

Overexpression of PAI1 increased *Col4a1*, *Fibronectin*, *ICAM1*, *Timp2*, and *Vimentin* in both parental and resistant cells of SKBR3 (Figure 3A) and HCC1954 (Figure 3B), while silence of PAI1 decreased in both parental and resistant in SKBR3 (Figure 3C) and HCC1954 (Figure 3D).

### 3.3. PAI1 Regulates Cell Morphology in Parental and Resistant HER2-Positive Cell Lines

Epithelial to mesenchymal transition is one of the markers to track and evaluate migratory characteristics in cell morphology. β-actin is the main component of the actin cytoskeleton [23]. Tracking actin proteins with different techniques, such as immunofluorescence, allows us to understand cell morphology. In our study, β-actin was analysed by immunofluorescence to observe PAI1 expression on cell morphology in trastuzumab-resistant SKBR3 and HCC1954 cell lines. The comparison was made between parental and resistant cells, as well as between PAI1-overexpressed and silenced cells.

**Figure 4:**
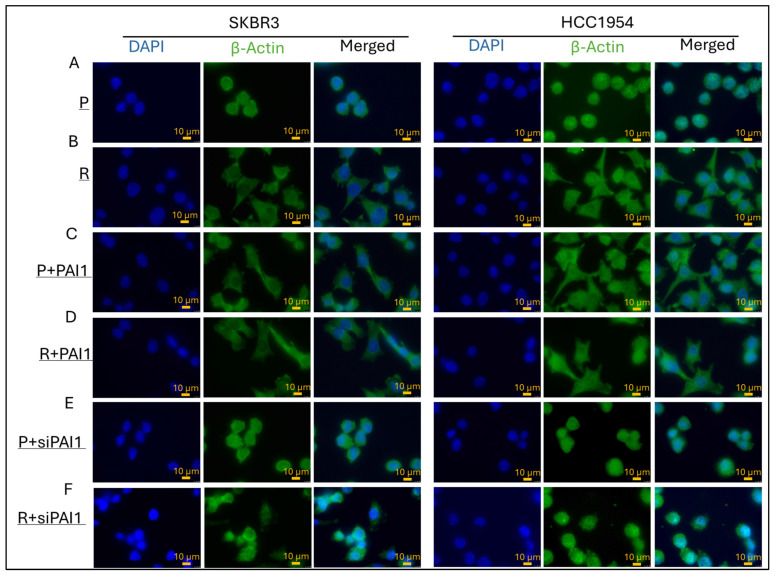
PAI1 regulates cell morphology in parental and resistant SKBR3 and HCC1954 cell lines. Immunofluorescence staining of parental and resistant SKBR3 and HCC1954 cell lines, DAPI indicates nucleus, green is β-Actin. Immunofluorescence of (**A**) parental cells showed epithelial and (**B**) resistant cells showed mesenchymal morphology in SKBR3 and HCC1954. (**C**) Overexpression of PAI1 in parental cells and (**D**) resistant cells showed mesenchymal morphology in SKBR3 and HCC1954. (**E**) Silencing of PAI1 in parental and (**F**) resistant cells showed epithelial morphology. Images are representative of three independent experiments. Scale bar 10 μm.

In SKBR3 and HCC1954 parental cell lines, an epithelial morphology with apical-basal polarity was observed (Figure 4A), while resistant cells exhibited a mesenchymal morphology with back–front polarity (Figure 4B). Overexpression of PAI1 in parental cells resulted in a transformation from epithelial to mesenchymal morphology in both SKBR3 and HCC1954 cell lines (Figure 4C), whereas resistant cells maintained their mesenchymal morphology (Figure 4D). Silencing of PAI1 in parental cells did not alter their epithelial morphology (Figure 4E). However, in resistant cells, the silencing of PAI1 resulted in a transformation from mesenchymal to epithelial morphology (Figure 4F) in both SKBR3 and HCC1954 cell lines.

### 3.4. Synergistic Effect of Trastuzumab+Aleplasinin on HER2-Positive Breast Cancer Cells

**Figure 5:**
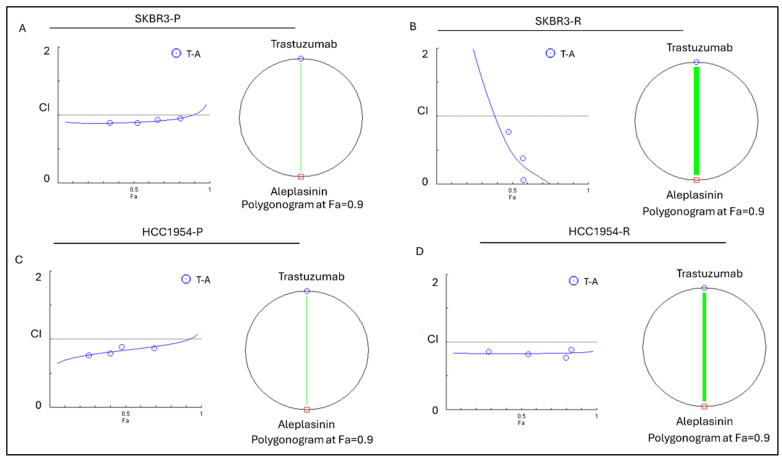
Trastuzumab and aleplasinin combination therapy results for combination index plot and polygonogram for SKBR3-P, SKBR3-R and HCC1954-P, HCC1954-R. The green line with specific thickness in (**A**) SKBR3-P polygonogram indicates a nearly additive effect; (**B**) SKBR3-R indicates very strong synergism; (**C**) HCC1954-P polygonogram indicates a nearly additive effect; (**D**) HCC1954-R indicates very strong synergism. Fa: Fraction affected; Fa = 0.9 means 90% cell death; P: parental; R: resistant; CI: combination index; CI > 1.1 represents antagonism; CI < 0.9 represents synergism; CI 0.9–1.1 represents an additive effect.

To assess the combined effect of the drugs on cells, the results were obtained using five concentration points at a 1:1 ratio for each drug: 0.25-fold, 0.5-fold, 1-fold, 2-fold, and 4-fold of the IC50 values for each individual drug. The IC50 values used in the experiments are listed in Table 1. The combination effect was evaluated by calculating the combination index (CI) using the median effect principle established by Chou and Talalay [18,24]. The type of line in the polygonogram shows the quantitation of synergism (Figure 5). The results showed that the trastuzumab+aleplasinin drug combination is nearly additive in SKBR3-P and HCC1954-P cell lines, very strongly synergistic in SKBR3-R, and synergistic in HCC1954-R cell line (Table 3).

### 3.5. Trastzumab+Aleplasinin Combination Decrease PAI1 Expression

To observe the short- and long-term effects of aleplasinin monotherapy and the combination therapy of trastuzumab and aleplasinin on PAI1 expression, cells were collected on days 2, 4, 10, and 15. The cells were treated with drugs daily at a concentration of IC50/4. DMSO was used as a control, and no changes were observed between the days.

**Figure 6:**
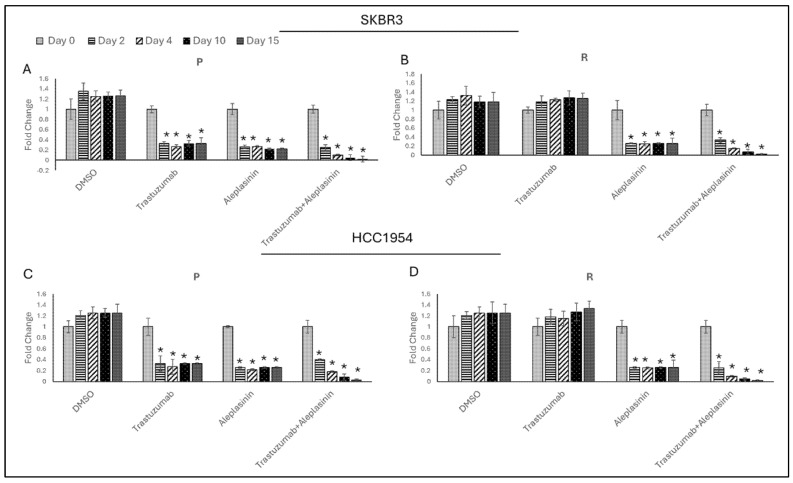
Trastuzumab+cilengitide combination decreased *PAI1* expression in parental and resistant SKBR3 and HCC1954 cell lines in both the short term and the long term. Trastuzumab+aleplasinin therapy decreased *PAI1* expression on day 2 and continued to decrease on day 4, day 10, and day 15 in (**A**) parental SKBR3, (**B**) resistant SKBR3, (**C**) parental HCC1954, and (**D**) resistant HCC1954 cells treated with DMSO, trastuzumab, aleplasinin as monotherapies, and trastuzumab+aleplasinin as combination therapy. Two-way ANOVA variation test and Tukey post hoc test were used. * *p* ≤ 0.05, n = 3 ± SD.

In SKBR3 and HCC1954 parental cell lines, both trastuzumab monotherapy and aleplasinin monotherapy decreased PAI1 expression on day 2, with levels remaining unchanged on days 4, 10, and 15 (Figure 6A,C). In SKBR3 and HCC1954 resistant cells, aleplasinin decreased PAI1 expression on day 2, and levels remained unchanged on days 4, 10, and 15, like the parental cells. However, trastuzumab did not affect PAI1 levels on any day (Figure 6B,D).

Interestingly, the combination therapy of trastuzumab and aleplasinin resulted in a decrease in PAI1 levels on day 2, with a significant and continued decrease observed on days 4, 10, and 15 in both SKBR3 and HCC1954 parental and resistant cells.

### 3.6. Trastzumab+Aleplasinin Combination Decrease Migration Responsive Marker Expressions

To observe PAI1 inhibition on migration in trastuzumab resistance, PAI1 inhibitor aleplasinin was used as monotherapy, and combination with trastuzumab and migration marker expressions were analysed 24 h after treatment in SKBR3 and HCC1954 parental and resistant cell lines.

**Figure 7:**
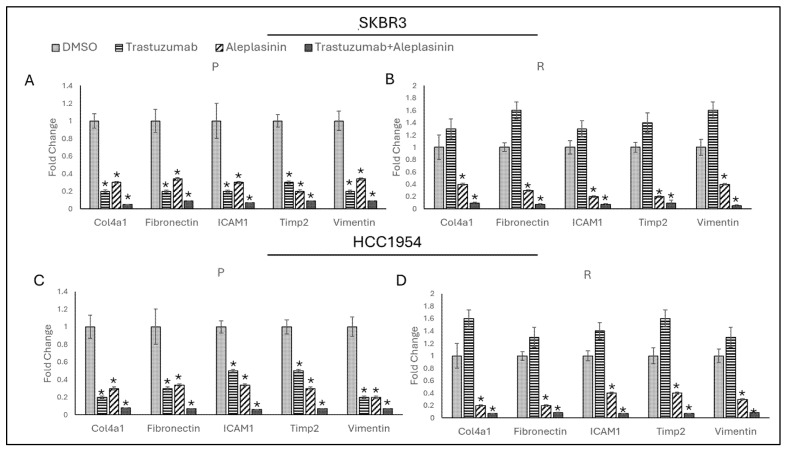
Trastuzumab+cilengitide combination decreased migration marker expressions in parental and resistant SKBR3 and HCC1954 cell lines. Trastuzumab and aleplasinin, both as monotherapies and in combination, decreased migration-responsive gene expressions in (**A**) SKBR3 and (**C**) HCC1954 parental cells; aleplasinin monotherapy and combination with trastuzumab decreased migration-responsive gene expressions in (**B**) SKBR3 and (**D**) HCC1954 resistant cells. P: parental, R: resistant. Two-way ANOVA variation test and Tukey post hoc test were used. * *p* ≤ 0.05, n = 3 ± SD.

It was observed that trastuzumab and aleplasinin as monotherapy and combination decreased *Col4a1*, *Fibronectin*, *ICAM1*, *Timp2,* and *Vimentin* expressions in SKBR3 and HCC1954 parental cell lines (Figure 7A,C). Trastuzumab monotherapy did not reduce any of the migration marker expressions. However, its combination with aleplasinin and aleplasinin monotherapy decreased *Col4a1*, *Fibronectin*, *ICAM1*, *Timp2*, and *Vimentin* expressions in SKBR3- and HCC1954-resistant cell lines.

## 4. Discussion

Trastuzumab resistance remains a significant obstacle in the management of HER2-positive breast cancer, necessitating a deeper understanding of the underlying molecular mechanisms to develop effective therapeutic strategies. The role of the TGF-β pathway in HER2-positive breast cancer was known, but its involvement in trastuzumab resistance was not well understood. In our study, we first focused on TGF-β responsive gene expressions in mediating resistance to trastuzumab.

The TGF-β pathway is known for its dual role in cancer progression, acting as a tumour suppressor in the early stages and a promoter of tumour aggressiveness in later stages. In a previous heatmap analysis, the expression of TGF-β responsive genes was determined in A549 (lung adenocarcinoma) and HPL1D (lung epithelial cell line). It was observed that *WWP1*, *CARM1*, *RASGRP1*, *THBS1*, *KCTD5*, *SGCA*, *EIF3S6*, *MCAM*, *FXR2*, *MTMR3*, *SOCS3*, *SLC2A4RG*, *MMP2*, *MMP9*, *HSP47*, and *PAI1* exhibited increased expression in HER2-positive drug-resistant cells.

However, the expressions of *NTSC2* and *ALAD* increased in HER2-positive trastuzumab-resistant cells while significantly decreasing in A549 and HPL1D cells. These findings contribute to the understanding of their potential as TGF-β responsive genes, except for NTSC2 and ALAD. Our analysis of TGF-β responsive genes revealed a striking upregulation of plasminogen activator inhibitor-1 (PAI1) among these genes in trastuzumab-resistant HER2-positive breast cancer cells. PAI1, a key regulator of extracellular matrix remodelling and proteolytic activities, emerged as a critical mediator of resistance mechanisms.

The marked upregulation of *PAI1* in trastuzumab-resistant SKBR3 and HCC1954 cells emphasizes its potential as a key player in resistance mechanisms. In previous studies, the role of PAI1 in cancer was associated with metastasis. For example, PAI-1 secreted by breast cancer cells activated PLOD2 in cancer-associated adipocytes (CAAs), facilitating the reorganisation of collagen into linear structures that promote cancer cell migration and metastasis. Targeting PAI-1 with inhibitors like tiplaxtinin reversed these effects, suggesting PAI-1 as a promising therapeutic target to inhibit cancer progression and disrupt the stromal collagen network conducive to metastasis [25]. However, the role of PAI1 in trastuzumab-resistant HER2-positive breast cancer remains unknown.

The robust increase in *PAI1* levels, observed as a 25-fold increase in SKBR3 and a 40-fold increase in HCC1954-resistant cells, suggests that PAI1 is not merely a bystander but a critical driver of the resistance phenotype.

The overexpression and silencing experiments further corroborate PAI1’s influence on cellular behaviour. Overexpression of PAI1 in both parental and resistant cells resulted in increased expression of migration markers such as *Col4a1*, *Fibronectin*, *ICAM1*, *Timp2*, and *Vimentin*, thereby promoting a migratory phenotype. Conversely, silencing PAI1 significantly reduced the expression of these markers, indicating its essential role in maintaining the migratory and invasive characteristics of resistant cells. This dual regulatory capacity of PAI1 highlights its potential as a therapeutic target; modulating its expression could effectively impair the metastatic potential of resistant cancer cells.

Cell morphology analysis via immunofluorescence provided further insights into the impact of PAI1 on EMT. Parental cells exhibited typical epithelial morphology, while resistant cells displayed mesenchymal characteristics indicative of enhanced migratory capacity. Overexpression of PAI1 induced a mesenchymal phenotype in parental cells while silencing PAI1 in resistant cells reversed their morphology to an epithelial state. These observations suggest that PAI1 not only promotes migration at the molecular level but also drives the phenotypic changes associated with EMT, a critical process in cancer metastasis.

It is well-documented that patients develop resistance to HER2-targeting drugs within a year [26]. Initially, these drugs block protein expression, but over time, cells adapt by increasing the copy number of the target protein-coding gene or altering its alternative splicing to restore the blocked protein expression [4]. To replicate this mechanism, resistant cells were generated and treated with trastuzumab and aleplasinin, both as monotherapies and in combination, for 2, 4, 10, and 15 days.

This experiment aimed to demonstrate that, at least within 15 days, resistance does not develop to the combination of trastuzumab and aleplasinin. It was observed that aleplasinin alone did not alter PAI1 gene expression over the subsequent days. However, the combination treatment of trastuzumab and aleplasinin proved to be more effective at silencing PAI1 gene expression than the inhibitor alone. This enhanced efficacy is likely attributed to the synergistic effect, which downregulates PAI1-associated pathways.

Furthermore, targeting PAI1 with aleplasinin effectively attenuated PAI1 expression, thereby reducing the migratory potential of trastuzumab-resistant cells. Notably, the combination treatment of trastuzumab and aleplasinin significantly suppressed cell migration and enhanced therapeutic efficacy compared to trastuzumab monotherapy alone.

Furthermore, mechanistic insights revealed that aleplasinin-mediated inhibition of PAI1 not only inhibited cell migration but also sensitised HER2-positive breast cancer cells to trastuzumab, suggesting a dual benefit of overcoming resistance and enhancing drug efficacy.

Overall, compelling evidence was provided by this study supporting the therapeutic potential of combining trastuzumab with PAI1 inhibition using aleplasinin in HER2-positive breast cancer. Combining trastuzumab with aleplasinin therapy could be particularly advantageous for patients with HER2-positive breast cancer who develop resistance to trastuzumab. By targeting PAI1, this combination may prevent or delay resistance, thus improving patient outcomes. This strategy could be incorporated into existing treatment regimens to enhance trastuzumab’s effectiveness and achieve a more durable response. Although PAI1 is known to regulate migration markers, the precise molecular mechanisms remain unclear and need further investigation. Animal studies and clinical trials are required to determine optimal dosing, scheduling, and biomarkers, as well as to evaluate long-term effects and potential resistance. Despite encouraging results, further research and clinical validation are essential to confirm the therapeutic potential and safety of targeting PAI1 in combination with trastuzumab for HER2-positive breast cancer.

## 5. Conclusions

The critical role of PAI1 in mediating migration and therapeutic response in trastuzumab-resistant HER2-positive breast cancer cells has been highlighted by our study. It was demonstrated that PAI1 expression is significantly upregulated in trastuzumab-resistant cell lines SKBR3 and HCC1954, with an increase in migration markers and TGF-β responsive genes observed. Cell morphology and migration capabilities were influenced by PAI1 overexpression or silencing, underscoring its regulatory role in cancer progression. Importantly, it was suggested that combining trastuzumab with the PAI1 inhibitor aleplasinin effectively reduces PAI1 expression, presenting a promising therapeutic strategy to overcome trastuzumab resistance in HER2-positive breast cancer. These insights contribute to ongoing efforts to enhance treatment efficacy and patient outcomes in this challenging disease context.

## Figures and Tables

**Table 1:** IC_50_ levels of aleplasinin for SKBR3 and HCC1954.

Cell Lines	p-IC_50_ (μM)	R-IC_50_ (μM)
SKBR3	7.5 ± 0.6	6.4 ± 0.6
HCC1954	10.2 ± 0.8	12.5 ± 1.1

**Table 2:** Real-time PCR primers used in experiments.

Gene Name	Sequence	Product Length
WWP1	F: GCAGCTCATCTCCAACCATAG	201 bp
	R: GAGACGGAGATGAAGGTGTG	
CARM1	F: CAGTTTTATGGCTACCTGTCCC	150 bp
	R: AAACGACAGGATCCCAGAGC	
RASGM1	F: AAATCAATGCCCGTGACTGG	166 bp
	R: TCAGAGAACGATATCCTCCGG	
THBS1	F: GCTCTACCAGTGTCCTCCTC	213 bp
	R: TCTCTTCAGTCACTTTGCGG	
KCDT1	F: AGGAGTGTTGGAGGAAGCAG	193 bp
	R: CAACTGCTCGAACTTCCAGC	
SGCA	F: CAGGTCATTGAGGTCACAGC	183 bp
	R: CCCCAAGGCTGAGAGGAAG	
MCAM	F: ACTGGTTTTCTGTCCACAAGG	203 bp
	R: GATGCGGTACTCCTGGGAC	
FXR2	F: GGGGATGAAGTGGAGGTTTATTC	177 bp
	R: AAGGGGATTGGGATTAACTGG	
MTMR3	F: TTCCCAGGAAGCAGCTGATC	249 bp
	R: ACTGACACCTGATAACTTTGCAG	
SOCS3	F: GAGAGCGGCTTCTACTGGAG	162 bp
	R: CTGGATGCGCAGGTTCTTG	
SLC2A4	F: AGAGCCACCCCAGGAAAAG	225 bp
	R: CGGAGAGGACTGGTCACTG	
MMP2	F: CAGGATCATTGGCTACACACC	151 bp
	R: CCAGCGGCCAAAGTTGATC	
MMP9	F: TGGATCCAAAACTACTCGGAAG	250 bp
	R: CATCGTCGAAATGGGCGTC	
PAI1	F: AGACCGATTATTGACCGACC	210 bp
	R: CCAGTTGTGAGATCCGCTAC	
TIMP1	F: GACCACCTTATACCAGCGTTATG	183 bp
	R: CCATCCTGCAGTTTTCCAGC	
TGIF1	F: AGGTCTGTAACTGGTTCATCAAC	231 bp
	R: GAGACAGTGGCCTCCCTAG	
COL6A1	F: AAGTCTTCTCGGTGGCCATC	198 bp
	R: CGAAGGAGCAGCACACTTG	
AAK1	F: TGAGGGATCTTTCAGGGCAC	208 bp
	R: GGCAACAGCTTCACAGGTATC	
MAN1A1	F: ACAGTGGGGTCAACATCATG	155 bp
	R: TGCGGATCAAATGAGTCTCG	
NT5C2	F: TGCTGTGTACAAGTCCCCAG	184 bp
	R: RGAGGTTTCCATAGGCATCGAC	
IRS1	F: TCTTCTTCATCGAGGTGGGC	170 bp
	R: CCCCGACGATTGGCTCTTAC	
KCNIP3	F: GGGCTTTAAGAATGAGTGTCCC	151 bp
	R: AAAGTGGATGGCCCCGTTC	
C4BPA	F: TCTACAAACGATGCAGACACC	236 bp
	R: GCTGTGCCTTCCATTCCTG	
PSG1	F: TCACCTTACACCTGGAGACTC	159 bp
	R: AGCTGTGAGTCATAGGGAGG	
ALAD	F: AGAGTTCCCAAGGACGAGC	172 bp
	R: ATGCTCCGTTTTCACTCAGG	
FGD1	F: AGAAGTTTGAAAGAGAGCCTGTG	214 bp
	R: CTATCAATGCCGCTGTCCCG	
TFAP2B	F: GTCCAGTCAGTTGAAGATGCC	165 bp
	R: GACGGAGCAAAACACCTCG	
AHCYL1	F: CGCTGGTCTGCTTGTAACATC	175 bp
	R: CCCCATCATCCAGGATCATG	
HSPG2	F: AGATGGTTTATTTCCGAGCCC	181 bp
	R: AGCTCCTTGATGAACACCAC	
IL1RAP	F: CGACTATCACTTGGTATATGGGC	164 bp
	R: CCTGGTGAGATGAAACGTACG	
EIF3S6	F: GGACAAGCATGGTTTTAGGC	186 bp
	R: TGCATTAAGATTTCAGAGGCCAG	
HSP47	F: CGCCATGTTCTTCAAGCCAC	244 bp
	R: CTTTTCAAGGCGCTCGAGAG	
MIDORI	F: ACAGCGACGTCAGGTTCAC	169 bp
	R: GCATCTTCTTCTCGACACCTG	
GAPDH	F: ACAGTTGCCATGTAGACC	152 bp
	R: TTGAGCACAGGGTACTTTA	

**Table 3:** Trastuzumab and aleplasinin combination therapy results in CI value. P: parental; R: resistant; CI: combination index; CI > 1.1 represents antagonism; CI < 0.9 represents synergism; CI 0.9–1.1 represents an additive effect.

Cell Lines	Drug Combo CI Value	Description of Effect
SKBR3-P	1.06	Nearly additive
SKBR3-R	0.00	Very strong synergism
HCC1954-P	0.92	Nearly additive
HCC1954-R	0.49	Synergism

## Data Availability

The datasets and materials used and/or analyzed during the current study are available from the corresponding author upon reasonable request.

## References

[1] Murthy R.K., Loi S., Okines A., Paplomata E., Hamilton E., Hurvitz S.A., Lin N.U., Borges V., Abramson V., Anders C. (2020). Tucatinib, Trastuzumab, and Capecitabine for HER2-Positive Metastatic Breast Cancer. N. Engl. J. Med..

[2] Baselga J., Swain S.M. (2010). CLEOPATRA: A phase III evaluation of pertuzumab and trastuzumab for HER2-positive metastatic breast cancer. Clin. Breast Cancer.

[3] Swain S.M., Miles D., Kim S.B., Im Y.H., Im S.A., Semiglazov V., Ciruelos E., Schneeweiss A., Loi S., Monturus E. (2020). Pertuzumab, trastuzumab, and docetaxel for HER2-positive metastatic breast cancer (CLEOPATRA): End-of-study results from a double-blind, randomised, placebo-controlled, phase 3 study. Lancet Oncol..

[4] Luque-Cabal M., Garcia-Teijido P., Fernandez-Perez Y., Sanchez-Lorenzo L., Palacio-Vazquez I. (2016). Mechanisms Behind the Resistance to Trastuzumab in HER2-Amplified Breast Cancer and Strategies to Overcome It. Clin. Med. Insights Oncol..

[5] Modi S., Saura C., Yamashita T., Park Y.H., Kim S.B., Tamura K., Andre F., Iwata H., Ito Y., Tsurutani J. (2020). Trastuzumab Deruxtecan in Previously Treated HER2-Positive Breast Cancer. N. Engl. J. Med..

[6] Boz Er A.B., Er I. (2024). Targeting ITGβ3 to Overcome Trastuzumab Resistance through Epithelial–Mesenchymal Transition Regulation in HER2-Positive Breast Cancer. Int. J. Mol. Sci..

[7] Bang Y.J., Giaccone G., Im S.A., Oh D.Y., Bauer T.M., Nordstrom J.L., Li H., Chichili G.R., Moore P.A., Hong S. (2017). First-in-human phase 1 study of margetuximab (MGAH22), an Fc-modified chimeric monoclonal antibody, in patients with HER2-positive advanced solid tumors. Ann. Oncol..

[8] Vivekanandhan S., Knutson K.L. (2022). Resistance to Trastuzumab. Cancers.

[9] Shi Q., Huang F., Wang Y., Liu H., Deng H., Chen Y.G. (2024). HER2 phosphorylation induced by TGF-beta promotes mammary morphogenesis and breast cancer progression. J. Cell Biol..

[10] Ranganathan P., Agrawal A., Bhushan R., Chavalmane A.K., Kalathur R.K., Takahashi T., Kondaiah P. (2007). Expression profiling of genes regulated by TGF-beta: Differential regulation in normal and tumour cells. BMC Genom..

[11] Yan X., Xiong X., Chen Y.G. (2018). Feedback regulation of TGF-beta signaling. Acta Biochim. Biophys. Sin. (Shanghai).

[12] Samarakoon R., Higgins S.P., Higgins C.E., Higgins P.J. (2019). The TGF-beta1/p53/PAI-1 Signaling Axis in Vascular Senescence: Role of Caveolin-1. Biomolecules.

[13] Milenkovic J., Milojkovic M., Jevtovic Stoimenov T., Djindjic B., Miljkovic E. (2017). Mechanisms of plasminogen activator inhibitor 1 action in stromal remodeling and related diseases. Biomed. Pap. Med. Fac. Univ. Palacky. Olomouc Czech Repub..

[14] Dovnik N.F., Takac I. (2017). Prognostic significance of uPA/PAI-1 level, HER2 status, and traditional histologic factors for survival in node-negative breast cancer patients. Radiol. Oncol..

[15] Kokot A., Gadakh S., Saha I., Gajda E., Lazniewski M., Rakshit S., Sengupta K., Mollah A.F., Denkiewicz M., Gorczak K. (2024). Unveiling the Molecular Mechanism of Trastuzumab Resistance in SKBR3 and BT474 Cell Lines for HER2 Positive Breast Cancer. Curr. Issues Mol. Biol..

[16] Li H., Yuan W., Bin S., Wu G., Li P., Liu M., Yang J., Li X., Yang K., Gu H. (2020). Overcome trastuzumab resistance of breast cancer using anti-HER2 chimeric antigen receptor T cells and PD1 blockade. Am. J. Cancer Res..

[17] Boz Er A.B. (2024). Integrin β3 Reprogramming Stemness in HER2-Positive Breast Cancer Cell Lines. Biology.

[18] Chou T.-C. (2018). The combination index (CI < 1) as the definition of synergism and of synergy claims. Synergy.

[19] Sun Y., Vandenbriele C., Kauskot A., Verhamme P., Hoylaerts M.F., Wright G.J. (2015). A Human Platelet Receptor Protein Microarray Identifies the High Affinity Immunoglobulin E Receptor Subunit alpha (FcepsilonR1alpha) as an Activating Platelet Endothelium Aggregation Receptor 1 (PEAR1) Ligand. Mol. Cell. Proteom..

[20] Jun F., Hong J., Liu Q., Guo Y., Liao Y., Huang J., Wen S., Shen L. (2017). Epithelial membrane protein 3 regulates TGF-beta signaling activation in CD44-high glioblastoma. Oncotarget.

[21] Czekay R.P., Wilkins-Port C.E., Higgins S.P., Freytag J., Overstreet J.M., Klein R.M., Higgins C.E., Samarakoon R., Higgins P.J. (2011). PAI-1: An Integrator of Cell Signaling and Migration. Int. J. Cell Biol..

[22] Ciriza J., Garcia-Ojeda M.E. (2010). Expression of migration-related genes is progressively upregulated in murine Lineage-Sca-1+c-Kit+ population from the fetal to adult stages of development. Stem Cell Res. Ther..

[23] Lappalainen P., Kotila T., Jegou A., Romet-Lemonne G. (2022). Biochemical and mechanical regulation of actin dynamics. Nat. Rev. Mol. Cell Biol..

[24] Chou T.C. (2010). Drug combination studies and their synergy quantification using the Chou-Talalay method. Cancer Res..

[25] Wei X., Li S., He J., Du H., Liu Y., Yu W., Hu H., Han L., Wang C., Li H. (2019). Tumor-secreted PAI-1 promotes breast cancer metastasis via the induction of adipocyte-derived collagen remodeling. Cell Commun. Signal.

[26] Yoon J., Oh D.Y. (2024). HER2-targeted therapies beyond breast cancer—An update. Nat. Rev. Clin. Oncol..

